# Efficacy and prognosis of neoadjuvant chemotherapy in HER2 low-expressing breast cancer: a retrospective single-center study

**DOI:** 10.3389/fonc.2024.1454726

**Published:** 2024-09-25

**Authors:** Yarong Yao, Huifen Zhen

**Affiliations:** Department of Breast Surgery, Shanxi Bethune Hospital, Shanxi Academy of Medical Sciences, Tongji Shanxi Hospital, Third Hospital of Shanxi Medical University, Taiyuan, China

**Keywords:** breast cancer, low expression of human epidermal growth factor receptor 2, neoadjuvant chem-otherapy, pathological complete response, prognostic factors

## Abstract

**Purpose:**

Human epidermal growth factor receptor 2 (HER2) is vital for breast cancer prognosis. The aim of this study was to analyze the clinicopathological data of HER2-negative breast cancer patients receiving neoadjuvant chemotherapy and the associated factors affecting the pathological complete response rate (pCR) and prognosis.

**Methods:**

Clinical data of 173 patients with primary HER2-negative breast cancer, who initially received neoadjuvant chemotherapy followed by surgical treatment at the Breast Surgery Department of Bethune Hospital in Shanxi Province from January 2012 to December 2022, were collected.

**Results:**

Compared to HER2-0 patients, HER2-low patients had higher T staging (*p* = 0.008), higher Ki67 proliferation index (*p* < 0.001), lower N staging (*p* = 0.001), and lower pCR rate (*p* < 0.001). Univariate analysis revealed that T stage, TNM stage, HR status, HER2 status, and Ki67 are risk factors that affect the pCR rate in HER-2 negative. Multivariate analysis identified HR status as an independent predictor of pCR rate. Kaplan–Meier survival curves showed that menstrual status, N staging, T staging, TNM staging, and pCR status affected the prognosis of HER2-low breast cancer patients (*p* < 0.05).

**Conclusion:**

HER2-low breast cancer exhibits distinct biological behaviors, suggesting personalized treatment approaches.

## 1 Introduction

Human epidermal growth factor receptor 2 (HER2) is a critical prognostic and predictive biomarker for breast cancer. All newly diagnosed patients with primary or metastatic breast cancer should be tested for HER2 protein levels by immunohistochemistry (IHC) and/or gene expression by *in situ* hybridization (ISH) to guide clinical treatment (1–3). Currently, HER2-positive breast cancer is defined when HER2 expression is 3+ or 2+ IHC score, and gene amplification is detected by ISH. Patients with HER2-positive tumors can be treated with HER2 pathway-blocking drugs that have significantly improved the clinical outcomes of HER2-positive breast cancer (4). Strikingly, 10–20% of breast cancer tumors are HER2-positive, and 80–90% are HER2-negative (5, 6). Patients with HER2-low status - defined as IHC scores 2+ and 1+ with negative ISH were considered to be HER2-negative. This group of patients was similar to HER2 non-expressing (HER2-0) patients, wherein none could benefit from the anti-HER2 therapy. The results of clinical trials of new antibody-drug conjugates (ADCs), such as T-DXd, suggested that the beneficiary population includes HER2-positive patients; also, the drugs show efficacy in HER2-low breast cancers (7, 8). These findings indicated that anti-HER2 therapy based on ADCs is efficacious in patients with low HER2 expression, further confirming that this group is not equivalent to the HER2-0 group and may have unique biological behaviors and therapeutic strategies.

HER2-low breast cancers account for approximately 45%–55% of all breast cancers (9). Owing to the large proportion of patients with HER2-low breast cancer, developing precision medicine strategies and improving survival in these patients is an urgent requisite that requires knowledge of the clinical characteristics and prognosis of these patients. Hitherto, only a few studies have focused on the clinicopathological characteristics of individuals with low HER2 expression and the relevance and prognosis of factors influencing the pathological complete response (pCR) with neoadjuvant chemotherapy. Thus, the present study aimed to analyze the clinicopathological data of HER2-0 breast cancer patients receiving neoadjuvant chemotherapy, further analyze the relevant factors affecting the pCR and prognosis of HER2-low breast cancer, and provide a basis for clinical research and the development of novel targeted drugs for precise treatment.

## 2 Information and methods

### 2.1 Clinical data

Clinical data of 173 patients with primary HER2-negative breast cancer treated surgically after initial neoadjuvant chemotherapy at the Breast Surgery Department of Bethune Hospital in Shanxi Province from January 2012 to December 2022 were collected. All patients had completed a dose-dense anthracycline sequential paclitaxel-based neoadjuvant chemotherapy regimen.

The inclusion criteria were as follows: ① women > 18-years-old; ② all of them underwent hollow-core needle puncture to identify breast cancer and confirm HER2-negative expression by IHC before chemotherapy; ③ met the indications of the Chinese Association Against Cancer (CAC) Breast Cancer Diagnostic and Treatment Guidelines and Criteria for neoadjuvant chemotherapy; ④ received neoadjuvant chemotherapy and underwent surgery after completing the established cycles of chemotherapy; ⑤ provided complete clinical data.

The exclusion criteria were as follows: (1) male; (2) inflammation, bilateral, lactation, and pregnancy breast cancer; (3) combined with other severe complications and malignant tumors; (4) intolerant of and; (5) chemotherapy while receiving other breast cancer-related treatments; (6) chemotherapy without surgical treatment; (7) distant metastases.

Relevant clinical data of patients, including age, menstrual status, body mass index (BMI), axillary and clavicular region lymph node status, T stage, N stage, TNM stage, hormone receptor (HR) status, Ki67, and tumor pathology type, were collected. The present study was approved by the institutional internal Ethics Review Board.

### 2.2 Treatment programs

Eight-cycle ddEC-ddP regimen: epirubicin 100 mg/m^2^, cyclophosphamide 600 mg/m^2^, one cycle every 14 days, four cycles followed by sequential four cycles of paclitaxel 175 mg/m^2^, one cycle every 14 days.

### 2.3 Immunohistochemical assessment

According to the guidelines of the American Society of Clinical Oncology (ASCO) and the College of American Pathologists (CAP)^3^, IHC-based HER2-negative is defined as HER2 0 or HER2 1+ or HER2 2+ and fluorescence *in situ* hybridization (FISH) negative. HER2-low indicates that the IHC result was 1+ or 2+, and the FISH result was negative (10). HER2 was accessed using HER2 antibody 4B5 (Roche, Basel, Switzerland) and ISH-techniques and classified according to the ASCO/CAP guidelines available during the different time periods (1–3). Estrogen receptor (ER) positivity and progesterone receptor (PR) positivity were standardized as ≥ 1% positive staining, whereas HR positivity was defined as ER or PR positivity.

### 2.4 Efficacy and survival analysis

The main objective of this study was to investigate the factors influencing the efficacy and prognosis of neoadjuvant chemotherapy in patients with HER2-low breast cancer. pCR was defined as the absence of invasive cancer residue in the breast and axillary lymph nodes. Only residual carcinoma *in situ* was classified as pCR. Disease-free survival (DFS) was defined as the time from surgery to all types of disease progression (including local recurrence, distant metastasis, neoplastic tumors, or death due to tumors). Follow-up time was calculated from the definitive pathological diagnosis to follow-up for up to five years.

### 2.5 Statistical methods

Statistical analyses were performed using R 4.3.2 and SPSS 25 software. Quantitative data that conformed to normal distribution were expressed as mean, whereas qualitative data were expressed as number of cases (percentage) [n (%)]. Univariate analyses were performed using t-test, chi-square test, Fisher’s exact test, univariate logistic regression model, and univariate Cox proportional regression model. Multivariate analyses used logistic and Cox proportional regression models. Survival curves were plotted using the Kaplan–Meier method. The test threshold was defined as a = 0.05.

## 3 Results

### 3.1 Comparison of clinicopathological features of differential HER2 protein expression

**Figure 1:**
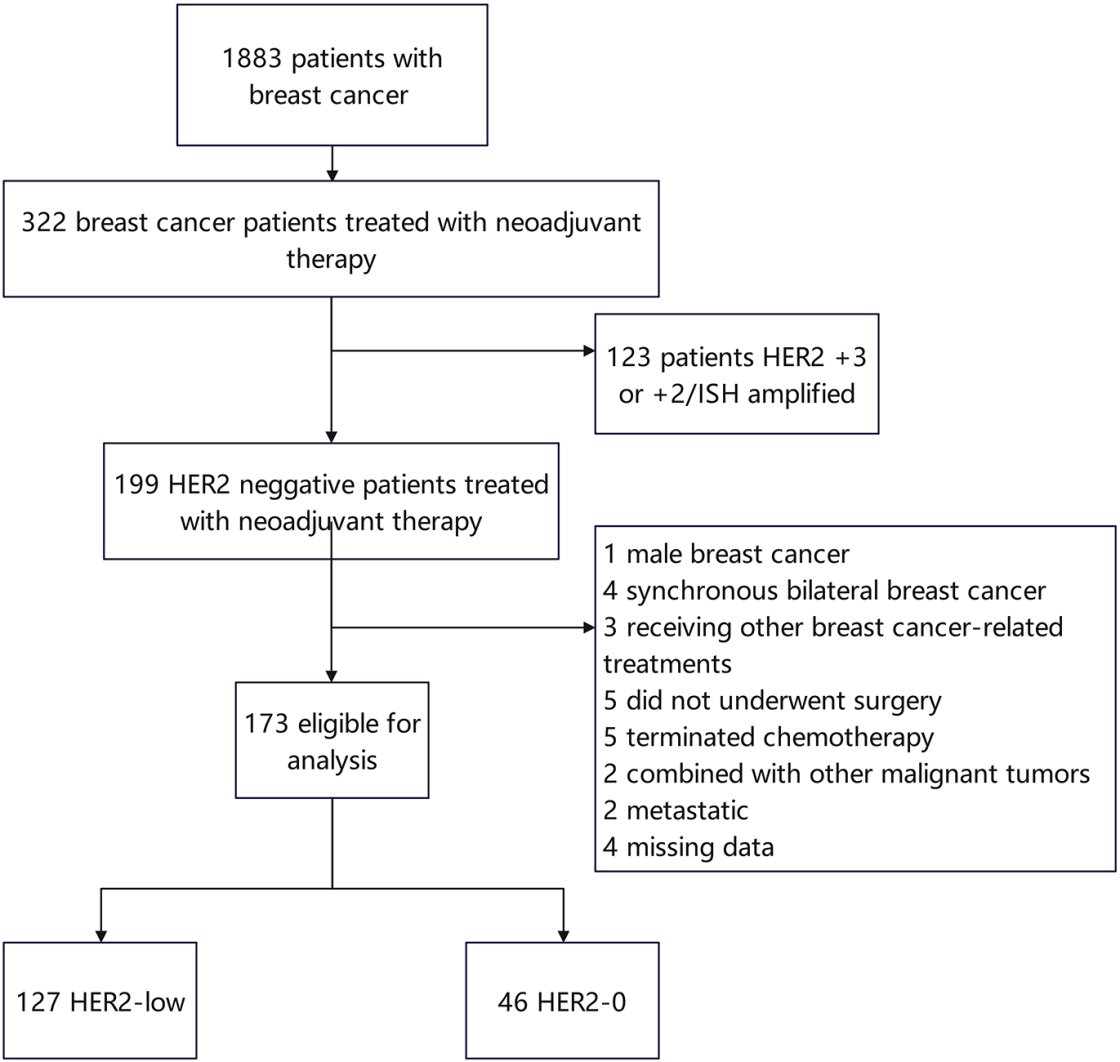
CONSORT diagram for study entry.

A total of 173 patients with HER2-negative breast cancer [including 127 (73.41%) patients with HER2-low and 46 (26.59%) patients with HER2-0] were recruited in this study (**Figure 1**). The median age of all patients was 50.09 years. 91/173 (52.60%) patients were in premenopausal status. Based on the IHC expression of HER2 (0, 1+, 2+), the patients were divided into HER2-0 and HER2-low to determine the differences in the clinicopathological features between the two subgroups. Compared to HER2-0 patients, HER2-low patients had higher T-stage (*p* = 0.008), higher Ki67 index (*p* < 0.001), lower N-stage (*p* = 0.001), and lower pCR rate (*p* < 0.001). The cohort comprised 79.19% luminal type (HR-positive) and 20.81% triple-negative breast cancer (TNBC) type (HR-negative) patients. A higher percentage of HR positivity was detected in the HER2-low group (*p* < 0.001) and was significantly frequent in the luminal type (83.9% *vs*. 16.1%) (**Table 1**).

**Table 1:** Clinical and pathological data of HER2-negative breast cancer patients.

Clinicopathological features	HER2		Total (N=173)	Statistics(t/χ^2^/R)	*p*
	HER2-0 (N=46)	HER2-low (N=127)			
Age	47.65 ± 10.84	50.98 ± 11.05	50.09 ± 11.06	-1.757	0.081
BMI	25.03 ± 3.77	25.69 ± 3.46	25.51 ± 3.54	-1.072	0.285
Menstruation				0.386	0.534
Premenopause	26(56.52%)	65(51.18%)	91(52.60%)		
Postmenopausal	20(43.48%)	62(48.82%)	82(47.40%)		
N stage				16.499	0.001
0	8(17.39%)	65(51.18%)	73(42.20%)		
I	21(45.65%)	36(28.35%)	57(32.95%)		
II	10(21.74%)	18(14.17%)	28(16.18%)		
III	7(15.22%)	8(6.30%)	15(8.67%)		
T stage				9.680	0.008
I-II	40(86.96%)	79(62.20%)	119(68.79%)		
III	4(8.70%)	29(22.83%)	33(19.08%)		
IV	2(4.35%)	19(14.96%)	21(12.14%)		
TNM stage				0.105	0.746
II	27(58.70%)	78(61.42%)	105(60.69%)		
III	19(41.30%)	49(38.58%)	68(39.31%)		
Supraclavicular lymph nodes				0.000	1.000
Yes	1(2.17%)	4(3.15%)	5(2.89%)		
No	45(97.83%)	123(96.85%)	168(97.11%)		
HR				37.407	0.000
–	24(52.17%)	12(9.45%)	36(20.81%)		
+	22(47.83%)	115(90.55%)	137(79.19%)		
Ki67				63.486	0.000
<20%	26(56.52%)	5(3.94%)	31(17.92%)		
≥20%	20(43.48%)	122(96.06%)	142(82.08%)		
Pathological types				0.221	0.638
IDC	43(93.48%)	121(95.28%)	164(94.80%)		
ILC	3(6.52%)	6(4.72%)	9(5.20%)		
pCR				27.053	0.000
CR	23(50.00%)	16(12.60%)	39(22.54%)		
PR	23(50.00%)	111(87.40%)	134(77.46%)		

### 3.2 Predictive factors for pCR

39/173 HER2-negative patients achieved pCR, with an overall rate of 22.54%. Univariate analysis identified T stage, TNM stage, HR status, HER2 status, and Ki67 as risk factors affecting the pCR rate. Moreover, patients with lower T stage, lower TNM stage, low Ki67, negative HR, and HER2-0 were likely to achieve pCR. Multifactorial analysis revealed that HR status has a significant effect on pCR and is an independent predictor of pCR rate: 13% in HR-positive (luminal type) patients compared to 58% in HR-negative (TNBC type) patients [odds ratio (OR) = 0.155, 95% confidence interval (95% CI): 0.052–0.464, *p* < 0.001]. The pCR rate of patients in the HER2-0 and HER2-low groups was 50% and 12.6%, respectively, indicating that the difference between the two groups was not statistically significant after multifactorial analysis (*p* = 0.086) (**Table 2**).

**Table 2:** Impact of clinicopathological characteristics on pCR rate.

Clinicopathological feature	Case n (N=173)	pCR n (%)	Univariate *p*-value	Multifactorial *p*-value	OR (95% CI)
Age	173		0.568	0.787	0.989(0.914, 1.070)
BMI	173		0.758	0.236	1.091(0.945,1.261)
Menstruation			0.851	0.924	
Premenopause	91	20(0.22)			Reference
Postmenopausal	82	19(0.23)			0.925(0.189,4.542)
N stage			0.072	0.269	
0	73	18(0.25)			Reference
I	57	17(0.30)			0.401(1.107,1.498)
II	28	3(0.11)			0.301(0.036,2.542)
III	15	1(0.07)			0.057(0.003,1.179)
T stage			0.046	0.441	
I-II	119	33(0.28)			Reference
III	33	3(0.09)			0.419(0.087,2.018)
IV	21	3(0.14)			1.497(0.251,8.922)
TNM stage			0.008	0.444	
II	105	31(0.30)			Reference
III	68	8(0.12)			0.576(0.140,2.371)
Supraclavicular lymph nodes			0.999	0.999	
No	168	39(0.23)			Reference
Yes	5	0(0.00)			0
HR			0.000	0.001	
–	36	21(0.58)			Reference
+	137	18(0.13)			0.155(0.052, 0.464)
Ki67			0.000	0.100	
<20%	31	20(0.65)			Reference
≥20%	142	19(0.13)			0.300(0.071,1.260)
Pathological types			0.432	0.526	
IDC	164	36(0.22)			Reference
ILC	9	3(0.33)			2.075(0.217,19.854)
HER2			0.000	0.086	
HER2-0	46	23(0.50)			Reference
HER2-low	127	16(0.13)			0.270(0.061,1.205)

The analysis of factors influencing the pCR rate in the HER2-low subgroup revealed HR status as its independent predictor. The pCR rate was 42% in the HR-negative group and 10% in the HR-positive group (OR = 0.080, 95% CI: 0.015–0.421, *p* = 0.003; **Table 3**).

**Table 3:** Impact of clinicopathological characteristics on pCR rate in the HER2-low patients.

Clinicopathological feature	Case n (N=127)	pCR n (%)	Univariate P value	Multifactorial P value	OR (95% CI)
Age	127		0.709	0.751	1.018(0.912, 1.137)
BMI	127		0.385	0.417	1.091(0.884,1.347)
Menstruation			0.919	0.928	
Premenopause	65	8(0.12)			Reference
Postmenopausal	62	8(0.13)			1.103(0.133,9.169)
N stage			0.372	0.326	
0	65	10(0.15)			Reference
I	36	3(0.08)			0.171(0.026,1.107)
II	18	3(0.17)			0.653(0.033,12.962)
III	8	0(0.00)			0
T stage			0.124	0.562	
I-II	79	13(0.16)			Reference
III	29	2(0.07)			0.289(0.030,2.791)
IV	19	1(0.05)			0.836(0.057,12.186)
TNM stage			0.240	0.170	
II	78	12(0.15)			Reference
III	49	4(0.08)			0.163(0.012,2.176)
Supraclavicular lymph nodes			0.999	0.999	
No	123	16(0.13)			Reference
Yes	4	0(0.00)			0
HR			0.004	0.003	
–	12	5(0.42)			Reference
+	115	11(0.10)			0.080(0.015,0.421)
Ki67			0.615	0.427	
<20%	5	1(0.20)			Reference
≥20%	122	15(0.12)			0.350(0.026,4.672)
Pathological types			0.140	0.022	
IDC	121	14(0.12)			Reference
ILC	6	2(0.33)			29.961(1.634,549.363)

### 3.3 Survival analysis of low HER2 expression

68/173 patients had recurrence, metastasis, or were deceased; 6/68 were HER2-0, and the remaining 62 were HER2-low patients. The five-year DFS was 51.18% for HER2-low patients and 86.96% for patients in the HER2-0 group, and the difference was statistically significant (*p* < 0.001). Univariate Cox regression model analysis of overall survival in patients with low HER2 expression revealed that age, menstrual status, N stage, T stage, TNM stage, and pCR were factors that affected the prognosis of breast cancer patients (*p* < 0.05). Indicators with statistically significant results from univariate analysis were included in the Cox regression model for multivariate analysis. The results showed that N-stage, T-stage, and pCR were independent factors for the prognosis of patients with low HER2 expression breast cancers (*p* < 0.05) (**Table 4**).

**Table 4:** Survival analysis in HER2-low patients.

Clinicopathological feature	Univariate P value	Multifactorial P value	OR (95% CI)
Age	0.012	0.909	0.998(0.961,1.036)
BMI	0.299		
Menstruation	0.019	0.269	1.597(0.696,3.664)
N stage	0.000	0.000	1.859(1.345,2.570)
T stage	0.000	0.000	3.113(2.088,4.640)
TNM stage	0.000	0.506	0.782(0.379,1.613)
Supraclavicular lymph nodes	0.471		
HR	0.973		
Ki67	0.184		
Pathological types	0.877		
pCR	0.031	0.027	0.347(0.136,0.886)

**Figure 2:**
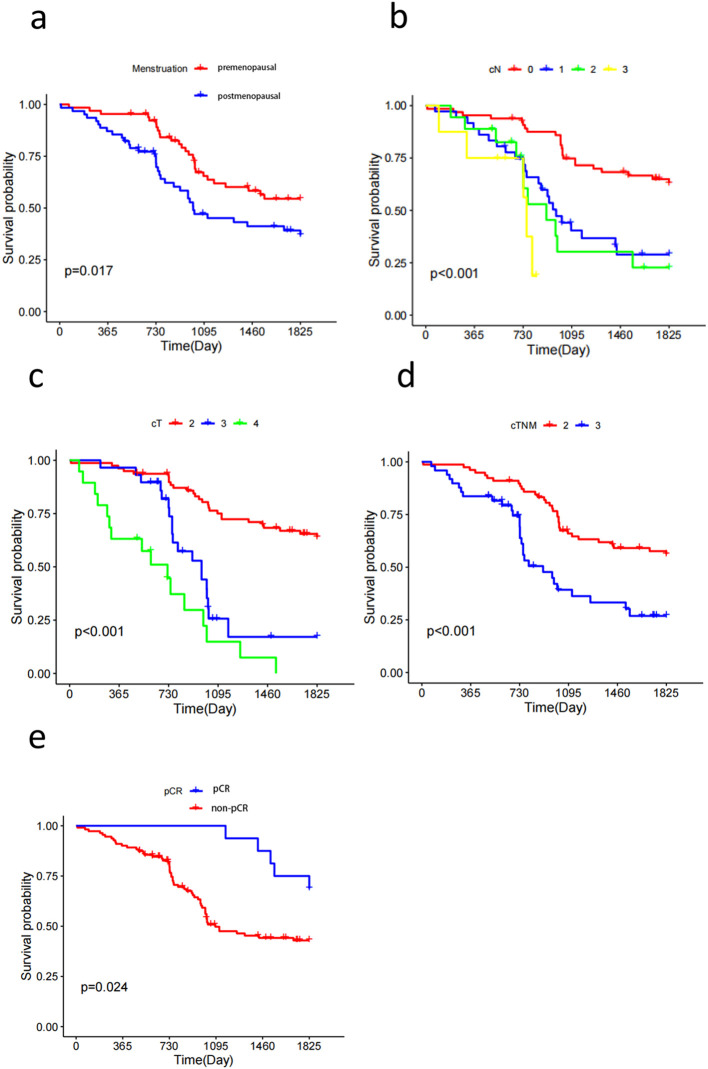
Kaplan-Meier survival curves show that Menstruation **(A)**, cN **(B)**, cT **(C)**, cTNM **(D)**, pCR **(E)** status are all factors affecting the prognosis of patients with Her2-low breast cancer.

Survival curves were plotted using the Kaplan–Meier method, and the results showed that menstrual status, N stage, T stage, TNM stage, and pCR factors affected the prognosis of HER2-low breast cancer (*p* < 0.05). The five-year DFS of premenopausal and postmenopausal patients was 58.5% and 43.5%, respectively (*p* = 0.017) (**Figure 2A**). The five-year DFS of the N0, N1, N2, and N3 groups was 64.6%, 36.1%, 38.9%, and 37.5%, respectively (*p* < 0.01) (**Figure 2B**). The five-year DFS of the T2, T3, and T4 groups was 65.8%, 34.5%, and 15.8%, respectively (*p* < 0.01) (**Figure 2C**). The five-year DFS reached 57.7% and 40.8% in TNM stages II and III, respectively (*p* < 0.01) (**Figure 2D**). Furthermore, the five-year DFS of patients with low HER2 expression who achieved pCR and those who were non-pCR after neoadjuvant therapy was 71.1% and 42.7%, respectively (*p* = 0.024) (**Figure 2E**).

## 4 Discussion

Recently, HER2 low-expressing breast cancer has gained increasing attention with the development of novel anti-HER2 targeted agents. HER2-low early-stage breast cancer appears to be a distinct biological entity. Further analysis revealed that factors such as menstrual status, N stage, T stage, TNM stage, and pCR affect the prognosis of patients with HER2-low breast cancer.

Previous studies have reported different clinical features of HER2-low breast cancer. Patients with low HER2 expression were often lymph node-positive. In addition, ductal histology revealed higher tumor grading and proliferation (11) characterized by the clinicopathological characteristics of breast cancers with varied levels of HER2 protein. Patients stratified according to HER2 status had similar age, menopausal status, and pathological stage distributions; however, statistical differences were noted in their histological types, histological grades, HR status, and Ki67 expression levels. Denkert et al. collected data of 2,310 patients with HER2-0 primary breast cancer from four prospective neoadjuvant clinical trials (12) and observed distinct clinical features of HER2-low breast cancer, such as significantly higher HR positivity, significantly lower Ki-67 expression, and lower pCR with neoadjuvant chemotherapy but longer overall survival. Another study (13) demonstrated that in patients with HR-positive breast cancer, HER2-low breast cancer was associated with fewer T4 tumors, high histologic grade, and negative lymphatic infiltration. In patients with TNBC, HER2-low was associated with a high lymph node ratio and positive lymphatic invasion. The current study showed that HER2-low patients had high T stage, high Ki67 index, low N stage, low pCR rate after neoadjuvant chemotherapy, high HR positivity, and high proportion of luminal type. Although the current findings were in line with previous studies, which concluded that HER2-low breast cancer has different clinicopathological features from HER2-0, there is no consensus on the specific manifestations.

The present study also analyzed the factors influencing the efficacy of neoadjuvant chemotherapy in HER2-negative patients. HR status was an independent predictor of pCR rate, whereas differential HER2 protein expression did not affect the pCR rate in multivariate analysis. In our study, we found that among all patients with HER2-negative breast cancer undergoing neoadjuvant treatment, HR-negative patients exhibited a higher pCR rate, which is consistent with previous research findings. For HR-positive, HER2-negative patients, chemotherapy can reduce the size of HR-positive tumors, aiding in optimizing surgical options. However, compared to histological types with higher proliferation rates, such as triple-negative breast cancer, it is more challenging for HR-positive breast cancer to achieve pathological complete response. On the other hand, univariate analysis revealed a pCR rate of 12.6% in HER2-low patients and 50% in HER2-0 patients (*p* < 0.001); however, the difference was not statistically significant after multivariate analysis (*p* = 0.086). Our study also suggested that HER2-low is less likely to achieve pCR in patients with HER2-0 breast cancer, consistent with previous findings (12, 14). Denkert et al. showed that HER2-low-positive tumors had a significantly lower pCR rate than HER2-0 tumors [321/1098 (29.2%) *vs*. 473/1212 (39.0%), *p* = 0.0002) (12). Another study found that HER2-low patients had significantly lower pCR rates with neoadjuvant chemotherapy than HER2-0 patients (15.9% *vs*. 37.5%, *p* = 0.042) (14). HER2-low tumors are frequently HR-positive (9), which was confirmed in our cohort with 79.19% of HR-positive tumors. This high proportion of HR-positive breast cancer explains our low pCR rate of 22.54%, which is lower than that expected for TNBC or HER2-positive tumors (15).

Finally, our study also analyzed the factors affecting the prognosis of HER2-low patients. Currently, the prognosis of HER2-overexpressing breast cancer is controversial. Denkert et al. concluded that patients with HER2-low-positive tumors had a significantly longer DFS than those with HER2-0 tumors [three-year rate: 83.4% (95% CI: 80.5–85.9) *vs*. 76.1% (95% CI: 72.9–79.0)] (12). Tarantino et al. (16) and other previous studies (17, 18) did not demonstrate marked differences in pCR rate or clinical outcomes between HER2-low and HER2-0 in multivariate analysis. Furthermore, we found that the five-year DFS was 51.18% in HER2-low patients and 86.96% in the HER2-0 group (*p* < 0.001). These conflicting results make distinguishing between HER2-low and HER2-0 difficult when using the traditional HER2 scoring system. The HER2 assay identified patients with HER2 overexpression tumors who benefited from trastuzumab; however, it has a limitation when evaluating the low levels of HER2 expression (19). This phenomenon could be ascribed to the wide variable incidence of HER2-low in retrospective studies: from 16.2% (20) to 64.4% (21). These findings suggested that precise diagnostic methods are essential to distinguish HER2-low breast cancer from HER2-0 breast cancer. The current study analyzed the factors affecting the prognosis of HER2-low patients; menstrual status, N-stage, T-stage, TNM-stage, and pCR affected the prognosis of patients with HER2-low breast cancer (*p* < 0.05). These results also provided a theoretical basis for evaluating the prognosis of HER2-low breast cancer patients after neoadjuvant chemotherapy.

Nevertheless, the present study has several limitations, such as the small sample size, its retrospective nature, absence of central pathological review and patient inclusion across a large time period, during which different guidelines for HER2 testing and interpretation were in use. It also provides some clues to the clinical and biological behaviors of the HER2-low breast cancer population that has a low pCR rate after neoadjuvant therapy and could benefit from new therapies. Currently, long-term follow-up of patients is limited; therefore, caution should be exercised while interpreting survival associations.

## 5 Conclusions

The current results have significant implications for future diagnostic concepts, a general understanding of the disease, and the development of new therapeutic strategies. The market launch of novel ADC drugs represented by T-DXd, especially the DESTINY-Breast 04 study results, has put forth a novel perspective on the diagnosis and treatment of HER2-low breast cancer. Both clinical and basic research have shown that HER2-low breast cancer exhibits distinct biological behaviors that may be associated with prognosis. This study also suggested that HER2-low breast cancer constitutes a significant proportion of the population and differs from HER2-0 in terms of clinicopathological features and prognostic characteristics, providing novel insights for individualized precision treatment of low HER2-expressing breast cancer patients.

## Data Availability

The original contributions presented in the study are included in the article/supplementary material. Further inquiries can be directed to the corresponding author.

## References

[1] Wolff AC Hammond MEH Schwartz JN Hagerty KL Allred DC Cote RJ. American Society of Clinical Oncology/College of American Pathologists guideline recommendations for human epidermal growth factor receptor 2 testing in breast cancer. J Clin Oncol. (2006) 25:118–45. doi: 10.1200/JCO.2006.09.2775 17159189

[2] Wolff AC Hammond ME Hicks DG Dowsett M McShane LM Allison KH. Recommendations for human epidermal growth factor receptor 2 testing in breast cancer: American Society of Clinical Oncology/College of American Pathologists clinical practice guideline update. J Clin Oncol. (2013) 31:3997–4013. doi: 10.1200/jco.2013.50.9984 24101045

[3] Wolff AC Somerfield MR Dowsett M Hammond MEH Hayes DF McShane LM. Human epidermal growth factor receptor 2 testing in breast cancer: ASCO-college of American pathologists guideline update. J Clin Oncol. (2023) 41:3867–72. doi: 10.1200/jco.22.02864 37284804

[4] Martínez-Sáez O Prat A. Current and future management of HER2-positive metastatic breast cancer. JCO Oncol Pract. (2021) 17:594–604. doi: 10.1200/op.21.00172 34077236

[5] Slamon DJ Clark GM Wong SG Levin WJ Ullrich A McGuire WL. Human breast cancer: correlation of relapse and survival with amplification of the HER-2/neu oncogene. Science. (1987) 235:177–82. doi: 10.1126/science.3798106 3798106

[6] Cronin KA Harlan LC Dodd KW Abrams JS Ballard-Barbash R. Population-based estimate of the prevalence of HER-2 positive breast cancer tumors for early stage patients in the US. Cancer Invest. (2010) 28:963–8. doi: 10.3109/07357907.2010.496759 PMC5094051 20690807

[7] Modi S Park H Murthy RK Iwata H Tamura K Tsurutani J. Antitumor activity and safety of trastuzumab deruxtecan in patients with HER2-low-expressing advanced breast cancer: results from a phase ib study. J Clin Oncol. (2020) 38:1887–96. doi: 10.1200/jco.19.02318 PMC7280051 32058843

[8] Modi S Jacot W Yamashita T Sohn J Vidal M Tokunaga E. Trastuzumab deruxtecan in previously treated HER2-low advanced breast cancer. N Engl J Med. (2022) 387:9–20. doi: 10.1056/NEJMoa2203690 35665782 PMC10561652

[9] Tarantino P Hamilton E Tolaney SM Cortes J Morganti S Ferraro E. HER2-low breast cancer: pathological and clinical landscape. J Clin Oncol. (2020) 38:1951–62. doi: 10.1200/jco.19.02488 32330069

[10] Xu B Hu X Feng J Geng C Jin F Li H. Chinese expert consensus on the clinical diagnosis and treatment of advanced breast cancer (2018). Cancer. (2020) 126 Suppl 16:3867–82. doi: 10.1002/cncr.32832 32710660

[11] Eggemann H Ignatov T Burger E Kantelhardt EJ Fettke F Thomssen C. Moderate HER2 expression as a prognostic factor in hormone receptor positive breast cancer. Endocr Relat Cancer. (2015) 22:725–33. doi: 10.1530/erc-15-0335 26187126

[12] Denkert C Seither F Schneeweiss A Link T Blohmer JU Just M. Clinical and molecular characteristics of HER2-low-positive breast cancer: pooled analysis of individual patient data from four prospective, neoadjuvant clinical trials. Lancet Oncol. (2021) 22:1151–61. doi: 10.1016/s1470-2045(21)00301-6 34252375

[13] Won HS Ahn J Kim Y Kim JS Song JY Kim HK. Clinical significance of HER2-low expression in early breast cancer: a nationwide study from the Korean Breast Cancer Society. Breast Cancer Res. (2022) 24:22. doi: 10.1186/s13058-022-01519-x 35307014 PMC8935777

[14] Zhang G Ren C Li C Wang Y Chen B Wen L. Distinct clinical and somatic mutational features of breast tumors with high-, low-, or non-expressing human epidermal growth factor receptor 2 status. BMC Med. (2022) 20:142. doi: 10.1186/s12916-022-02346-9 35484593 PMC9052533

[15] Spring LM Fell G Arfe A Sharma C Greenup R Reynolds KL. Pathologic complete response after neoadjuvant chemotherapy and impact on breast cancer recurrence and survival: A comprehensive meta-analysis. Clin Cancer Res. (2020) 26:2838–48. doi: 10.1158/1078-0432.ccr-19-3492 PMC7299787 32046998

[16] Tarantino P Jin Q Tayob N Jeselsohn RM Schnitt SJ Vincuilla J. Prognostic and biologic significance of ERBB2-low expression in early-stage breast cancer. JAMA Oncol. (2022) 8:1177–83. doi: 10.1001/jamaoncol.2022.2286 PMC9227690 35737367

[17] Schettini F Chic N Brasó-Maristany F Paré L Pascual T Conte B. Clinical, pathological, and PAM50 gene expression features of HER2-low breast cancer. NPJ Breast Cancer. (2021) 7:1. doi: 10.1038/s41523-020-00208-2 33397968 PMC7782714

[18] Hein A Hartkopf AD Emons J Lux MP Volz B Taran FA. Prognostic effect of low-level HER2 expression in patients with clinically negative HER2 status. Eur J Cancer. (2021) 155:1–12. doi: 10.1016/j.ejca.2021.06.033 34311211

[19] Moutafi M Robbins CJ Yaghoobi V Fernandez AI Martinez-Morilla S Xirou V. Quantitative measurement of HER2 expression to subclassify ERBB2 unamplified breast cancer. Lab Invest. (2022) 102:1101–8. doi: 10.1038/s41374-022-00804-9 35595825

[20] Jacot W Maran-Gonzalez A Massol O Sorbs C Mollevi C Guiu S. Prognostic value of HER2-low expression in non-metastatic triple-negative breast cancer and correlation with other biomarkers. Cancers (Basel). (2021) 13:6059. doi: 10.3390/cancers13236059 34885167 PMC8656488

[21] Horisawa N Adachi Y Takatsuka D Nozawa K Endo Y Ozaki Y. The frequency of low HER2 expression in breast cancer and a comparison of prognosis between patients with HER2-low and HER2-negative breast cancer by HR status. Breast Cancer. (2022) 29:234–41. doi: 10.1007/s12282-021-01303-3 34622383

